# CRISPR Disruption of scaRNA1 Reduces Pseudouridylation in Spliceosomal RNA U2 at U89 and Perturbs the Transcriptome in HEK293T Cells

**DOI:** 10.3390/cells14231882

**Published:** 2025-11-27

**Authors:** Amanda Gardner-Kay, Lynndy Le, Michael Filla, Nataliya Kibiryeva, James E. O’Brien, Douglas C. Bittel

**Affiliations:** 1 College of Bioscience, Kansas City University, Kansas City, MO 64106, USA; amanda.gardner-kay@kansascity.edu (A.G.-K.); lynndy.le@kansascity.edu (L.L.); mfilla@kansascity.edy (M.F.); nkibiryeva@kansascity.edu (N.K.); 2 Ward Family Heart Center, Children’s Mercy Hospital, Kansas City, MO 64108, USA; jobrien@cmh.edu

**Keywords:** noncoding RNA, spliceosome, scaRNA, snoRNA, alternative splicing

## Abstract

Small Cajal body-associated RNAs (scaRNAs) are essential for biochemical modification of spliceosomal RNAs and spliceosome function. Changes in scaRNA expression level have been associated with developmental issues, including cancer and congenital heart defects (CHDs), although the mechanism remains unclear. Small Cajal body-associated RNA 1 (scaRNA1) guides pseudouridylation at uridine 89 (Ψ89) of the spliceosomal RNA U2, a highly conserved modification that may be critical for spliceosome function. To investigate the role of scaRNA1 in splicing regulation, CRISPR-Cas9 genome editing was used to introduce targeted deletions in the scaRNA1 locus in HEK293T cells. Edited clones were identified by T7 endonuclease I assay and confirmed by Sanger sequencing. Pseudouridylation at Ψ89 was quantified using CMC-based reverse transcription followed by quantitative PCR, and global mRNA splicing alterations were assessed by RNA sequencing. Clones harboring scaRNA1 disruptions exhibited a significant reduction in Ψ89 pseudouridylation, consistent with impaired scaRNA1 function. Transcriptome analysis (of mRNA from two clones) revealed >300 protein coding genes with significant changes in transcript isoform level, including >100 genes related to RNA-binding activity. These results indicate that scaRNA1 disruption alters spliceosomal function and leads to substantial changes in mRNA splicing. The dysregulated splicing of RNA-binding proteins may impair RNA processing and gene expression programs required for normal development, providing new insight into how noncoding RNA dysfunction may contribute to developmental pathogenesis.

## 1. Introduction

Pre-mRNA splicing is an essential step in eukaryotic gene expression, catalyzed by the spliceosome—a dynamic ribonucleoprotein complex composed of five small nuclear RNAs (snRNAs) and numerous associated proteins [1]. This machinery not only facilitates the removal of introns and the ligation of exons but also governs alternative splicing (AS), a regulatory mechanism that enables the generation of multiple transcript isoforms from a single gene [1,2]. Through AS, the functional complexity of the transcriptome and proteome is greatly expanded, which play a pivotal role in cellular differentiation, tissue identity, and developmental signaling [3].

Accurate splicing depends on the post-transcriptional modifications of spliceosomal snRNAs, including 2′-O-methylation and pseudouridylation, which are concentrated in regions critical for RNA–RNA and RNA–protein interactions [4]. These modifications stabilize RNA’s secondary structure, enhance small nuclear ribonucleoprotein (snRNP) assembly, and promote catalytic efficiency [5,6,7]. Among the snRNAs, U2 is the most extensively modified, and pseudouridines are particularly enriched near the branch site recognition region—an area critical for proper splice site selection [4,8].

RNA modifications are introduced by two main mechanisms: site-specific enzymatic catalysis by proteins such as PUS1 and PUS7, and guide-RNA-directed modification via scaRNAs [9,10]. scaRNAs represent a specialized subclass of small nucleolar RNAs (snoRNAs) that localize to the Cajal body, a membraneless nuclear sub-organelle rich in RNAs and proteins involved in RNA maturation and the direct chemical modifications of spliceosomal snRNAs [11]. There are two subtypes of scaRNAs: Box H/ACA scaRNAs, which guide pseudouridylation, and Box C/D scaRNAs, which direct 2′-O-methylation, acting in coordination with accessory proteins like dyskerin and fibrillarin [10,12].

One such scaRNA, scaRNA1, guides pseudouridylation (designated here as Ψ) of the U2 snRNA at uridine 89 (Ψ89), a highly conserved residue within the branch site recognition region, critical for splice site selection and catalytic activity of the spliceosome [13]. The loss or dysregulation of site-specific pseudouridylation has been implicated in a range of pathologies, including neurodevelopmental disorders, malignancies, and congenital heart defects (CHDs), highlighting the functional significance of RNA-guided RNA modification [7,13,14]. Prior transcriptomic profiling of right ventricular myocardium of infants with Tetralogy of Fallot (TOF), a severe CHD marked by right ventricular outflow tract malformations, has revealed that scaRNA1 was among 12 scaRNAs that were significantly downregulated in the affected tissue [15]. Functional studies in cardiomyocytes revealed that knockdown of scaRNA1 along with another scaRNA, SNORD94, led to decreased U2 snRNA levels, widespread alternative splicing defects, and aberrant isoforms of cardiac regulatory genes, including GATA4, MBNL1/2, and NOTCH2 [13]. In contrast, the co-expression of both scaRNAs was sufficient to restore snRNA abundance and partially rescue normal splicing profiles and cardiomyocyte maturation [13]. These findings implicate scaRNA1-directed pseudouridylation as a key post-transcriptional regulator of cardiac development.

Despite these insights, the precise molecular consequences of isolated scaRNA1 loss remain incompletely defined. To address this, we employed CRISPR-Cas9 genome editing to introduce targeted edits within the scaRNA1 locus in human embryonic kidney (HEK293T) cells—a well-established model system in molecular genetic studies. Edited clones were identified by Sanger sequencing, and U2 pseudouridylation was assessed using CMC-based reverse transcription followed by quantitative PCR (as described by Nagasawa et al., 2020) [15]. To investigate transcriptome-wide effects, we performed RNA-seq and splicing analysis. Together, these approaches enabled a mechanistic dissection of scaRNA1 function, providing insight into how scaRNA-guided snRNA modifications influence pre-mRNA splicing that likely influences the composition of the transcriptome.

## 2. Materials and Methods

### 2.1. Guide RNA Design

**Figure 1:**
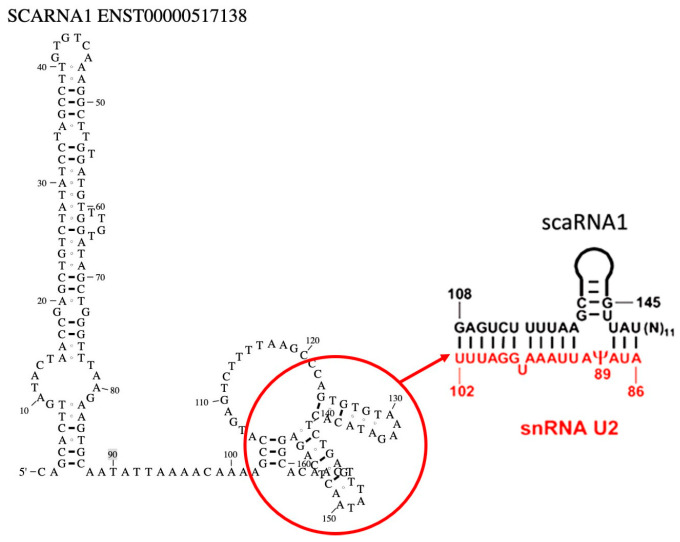
Predicted secondary structure of scaRNA1 and its base pairing with U2 snRNA at pseudouridylation site Ψ89. The left panel shows the predicted secondary structure of scaRNA1 (ENST00000517138). The region circled in red highlights the sequence that base pairs with snRNA U2 and the region targeted for editing. The right panel illustrates this base pairing interaction between scaRNA1 and U2 snRNA, guiding site-specific pseudouridylation at position uridine 89. Adapted from [17,18].

Guide RNAs (gRNAs) targeting human scaRNA1 (ENST00000517138, Ensembl (v115) were designed using the CRISPOR tool (http://crispor.tefor.net, accessed on 8 May 2024), which prioritizes candidates based on predicted on-target efficiency and off-target effects [16,17]. Two gRNAs were selected (Table 1) based on their positioning within the scaRNA1 region, known to base pair with snRNA U2 near the pseudouridylation site Ψ89 (Figure 1). Synthesized crRNAs and tracrRNAs were obtained from Integrated DNA Technologies (IDT, Coralville, IA, USA).

### 2.2. HEK293T Cell Culture and CRISPR-Cas9 Transfection

Human Embryonic Kidney 293T (HEK293T, CVCL0063, and ATCC) cells were maintained in T-25 flasks containing complete medium composed of Dulbecco’s Modified Eagle Medium (DMEM) (Gibco–Thermo Fisher Scientific, Waltham, MA, USA), 10% fetal bovine serum (FBS), and 1× penicillin–streptomycin at 37 °C with 5% CO_2_. Viability and cell counts were assessed using Trypan Blue exclusion and quantified with a Bio-Rad TC20 Automated Cell Counter (Hercules, CA, USA).

CRISPR-Cas9 transfections were performed using the TrueCut™ Cas9 Protein v2 with Lipofectamine CRISPR Max Transfection Reagent (Thermo Fisher Scientific), following the manufacturers protocol. HEK293T cells were seeded in 24-well plates at 4 × 10^4^ cells per well and grown to approximately 80% confluence. Experimental conditions included untreated cells, Cas9-only reagent control, and cells transfected with gRNA1, gRNA2, or both gRNAs. For the dual-gRNA condition, gRNA and reagent volumes were doubled to maintain molar equivalency. Cells were incubated at 37 °C with 5% CO_2_ for 72 h post-transfection prior to harvest, for downstream analyses.

To initially detect CRISPR-induced insertions or deletions (indels), we performed a T7 endonuclease I (T7E1) assay on cells populations using the Alt-R Genome Editing Detection Kit (IDT, Newark, NJ, USA) according to manufacturer’s protocol. Following the T7E1 assay assessment, CRISPR-edited single-cell clones were obtained by limiting the dilution in 96-well plates, starting at 1000 cells/mL.

### 2.3. DNA Isolation, Mutation Screening (Sanger Sequencing), and Validation by TIDE Analysis

Genomic DNA was extracted from HEK293T cells using the Direct Mouse Genotyping Kit (APExBIO, Houston, TX, USA), following the manufacturer’s protocol. DNA concentration and purity were assessed using a NanoDrop 2000 spectrophotometer (Thermo Fisher Scientific).

For the Sanger sequencing validation, first, we performed PCR amplification of the *scaRNA1* target region using gene-specific primers (Table 2) and the APExBIO Direct Mouse Genotyping Kit under standard cycling conditions.

The sequencing reaction was performed in both directions using the BigDye Terminator v3.1 sequencing Kit (Thermo Fisher Scientific) according to the company-provided protocol, and was visualized by SeqStudio Genetic Analyzer (Thermo Fisher Scientific).

Furthermore, we quantified the indels in sequence traces from wild-type and edited samples by Tracking of Indels using the Decomposition (TIDE) web tool (https://tide.nki.nl, accessed on 2 May 2025) with the following parameters: left boundary of 100 bp, decomposition window of 115–560 bp, indel size range of 2–27 bp, and *p*-value threshold of 0.001. The right boundary was set 10 bp upstream of the cut site. We only interpret results with R^2^ ≥ 0.90. Based on these evaluation steps, clones 6A1 and 3B2 were selected for further analysis.

### 2.4. RNA Extraction and cDNA Synthesis

Total RNA was extracted from the selected clones using the Trizol Reagent Phasemaker Tubes (Thermo Fisher Scientific) according to the manufacturer-provided protocol. Total RNA was treated with the DNase-free Kit (Thermo Fisher Scientific), following the manufacturer’s instructions and quantified by NanoDrop.

Complementary DNA (cDNA) was synthesized from 500 ng of the DNase-treated total RNA using the Maxima H minus First-Strand Synthesis Kit (Thermo Fisher Scientific) with random hexamer primers, following the manufacturer’s protocol.

### 2.5. qPCR for RNA Expression Analysis

To determine the impact of targeted CRISPR-Cas9 editing on *scaRNA1* expression, we performed qPCR analysis on the 6A1 HEK293T and 3B2 HEK293T cells harboring CRISPR-induced indels within the *scaRNA1* locus. qPCR was performed in triplicates using Maxima SYBR Green PCR MasterMix or TaqMan^TM^ Fast Advance Master Mix (Thermo Fisher Scientific) with the following primers and probe assays (Integrated DNA Technologies, Coralville, IA, USA) (Table 3). The quantitative PCR analysis included three technical replicates from three independent cDNA reactions. The statistical analysis used the Graph Pad Prism nested *t*-test.

The experiment was repeated three times with mean, standard error, and *p*-values calculated by Microsoft Excel.

The expression levels of *scaRNA1* and *PP1R8* were quantified and normalized to the reference gene *RN7SL*. The ΔΔCt method was used to calculate the relative fold change in *scaRNA1* expression compared to wild-type (WT) cells.

### 2.6. CMC Reverse Transcription Stop Assay for Pseudouridylation Detection

The pseudouridylation at *U2* snRNA position Ψ89 was measured using a CMC (N-cyclohexyl-N′-(4-methylmorpholinium)ethylcarbodiimide) reverse transcription stop assay [19,20], with matched CMC-treated RNA and untreated control RNA. Each sample was reverse transcribed, and qPCR was performed using Maxima SYBR Green Master Mix (Thermo Fisher Scientific) for the quantification of two amplicons: long fragment (108 bp)-spanning ψ89 and capturing RT stop (upstream forward and shared reverse primer pair); and short fragment (65 bp)—which served as an internal control and amplified beyond the ψ89 site (downstream forward and shared reverse primer pair) (Table 4).

The experiment was repeated three times and the pseudouridylation RT stop index (R) was defined from ΔCt values; higher R values indicate less pseudouridylation at *U2*-89. The R was calculated as follows:

$$R=\frac{2^{\left(average\;{\Delta Ct}_{control\;long\;fragment}-average\;{\Delta Ct}_{\;CMC\;treated\;long\;fragment}\right)}}{2^{\left(average\;{\Delta Ct}_{control\;short\;fragment}-average\;{\Delta Ct}_{\;CMC\;treated\;short\;fragment}\right)}}$$

To express pseudouridylation as a percentage, the ratio was converted using the following: Percent Pseudouridylation= 100×(1−R).

### 2.7. RNA-Seq Library Preparation and Analysis

RNA libraries were prepared using RNA obtained from the control HEK293T cells and two clones (3B2 and 6A1) using the Tecan Universal Plus mRNA-Seq kit and sequenced at the University of Kansas Medical Center Genome Sequencing Facility. Reads with a Phred quality score > 30 were aligned to hg38 with STAR 2.7.8a and quantified using Partek Flow 12.4.3 (Illumina, San Diego, CA, USA) with Ensemble 112 annotation. After TMM normalization, the differential transcript expression for protein coding genes was assessed by Poisson regression with FDR correction for multiple testing. Transcripts with adjusted *p*-value < 0.05 were further separated into two groups—upregulated (with fold change greater than 1.5) and downregulated (with fold change less than −1.5). Only genes that had transcripts in both groups, indicating potential isoform switch, were considered for Gene Ontology Enrichment Analysis.

## 3. Results

### 3.1. Edit of the Genomic Sequence of scaRNA1 Using CRISPR-Cas9

To model the observed reduction in the scaRNA1 expression as seen in the myocardium of infants with TOF [15], CRISPR-Cas9 gRNAs were designed to introduce targeted edits within the scaRNA1 genomic locus. These gRNAs were transfected into human embryonic kidney (HEK293T) cells to generate clonal cell lines harboring the intended genomic modification. Following transfection, genomic DNA was extracted from cell populations for the preliminary assessment of genome editing efficiency, and the scaRNA1 locus was amplified by PCR. Furthermore, PCR products were subjected to mismatch detection using the IDT Alt-R Genome Editing Detection Kit, which relies on T7 endonuclease I (T7EI) cleavage. T7EI specifically recognizes and cleaves DNA heteroduplexes formed during reannealing of wild-type and edited PCR products, producing characteristic cleavage bands detectable by gel electrophoresis.

**Figure 2:**
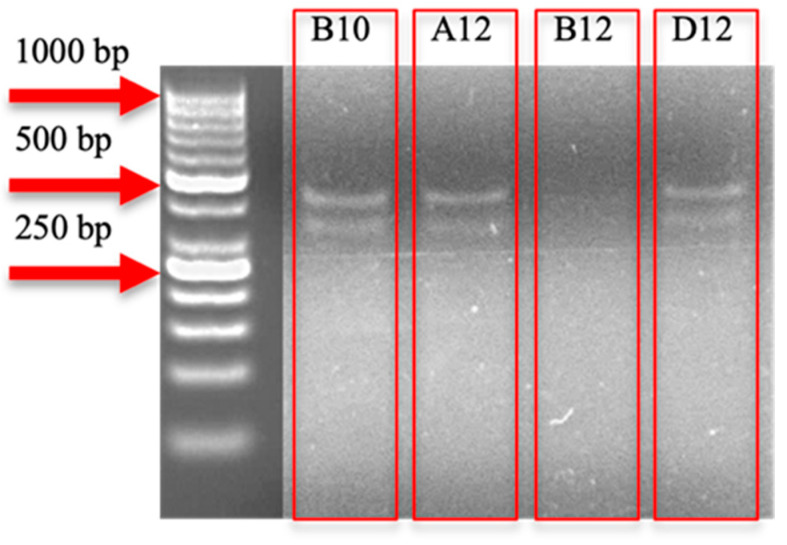
T7 endonuclease I assay detecting CRISPR-Cas9 editing in cell populations. A 2% agarose gel stained with ethidium bromide shows the results of a T7 endonuclease I (T7E1) assay used to detect CRISPR-Cas9-mediated genome editing in mixed cell populations. The DNA ladder in the leftmost lane includes size markers at 1000 bp, 500 bp, and 250 bp (indicated by red arrows). Lanes labeled B10, A12, and D12 show multiple bands, consistent with successful editing. In contrast, lane B12 lacks visible bands, indicating either a lack of detectable editing or a homozygous edit that does not generate heteroduplexes.

Figure 2 illustrates the T7EI digestion products indicative of successful CRISPR-Cas9-mediated editing for three mixed cell populations (B10, A12, and D12). Once a cell line exhibited preliminary evidence of genome editing via the T7EI assay, it was selected to produce a single-cell colony by limiting the dilution. To confirm the editing and to precisely characterize the introduced sequence alterations, PCR amplicons from candidate clones were analyzed using Sanger sequencing by a SeqStudio Genetic Analyzer (Thermo Fisher Scientific, Waltham, MA, USA). Resulting chromatograms were evaluated using SnapGene (v8.0.2), Benchling, and the TIDE algorithm to detect and quantify insertions or deletions (indels) at the targeted locus.

**Figure 3:**
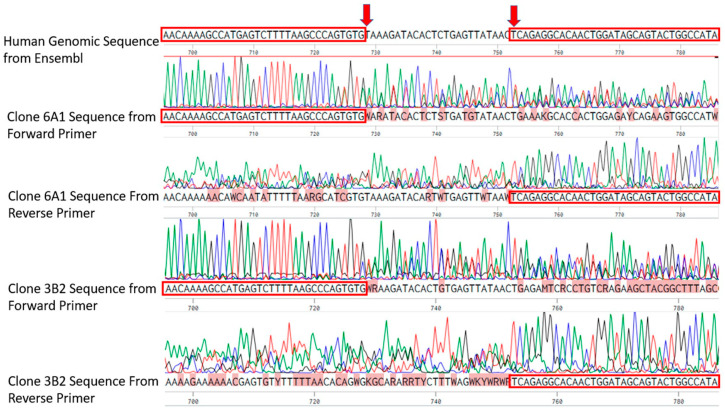
Human genomic sequence alignment to clone 6A1 sequencing results (from Benchling alignment tool). Clones 6A1 and 3B2 exhibit sequence ambiguity and overlapping peaks near the expected edit points based on the PAM sequences (red arrows). Red boxes show unambiguous sequences flanking each deletion site. Multiple ambiguous base calls are observed surrounding the cleavage sites, with increasing signal degradation and loss of clear sequence fidelity downstream, confirming successful editing at the targeted region.

Clone 6A1 and 3B2 displayed clear sequence divergence from the wild-type allele. As shown in Figure 3, overlapping chromatogram peaks at the predicted Cas9 cleavage site are consistent with heterozygous or mixed repair outcomes, indicating the presence of indels. These findings confirm the successful CRISPR-Cas9-mediated editing of the scaRNA1 locus in both clones and support their use in downstream functional analyses.

### 3.2. TIDE Analysis Reveals Distinct Clonal Editing Profiles

To quantify genome editing efficiency and characterize indel patterns at the scaRNA1 locus, we performed TIDE (Tracking of Indels by Decomposition) analysis on Sanger sequencing data from edited clones 6A1 and 3B2 [21]. Clone 6A1 exhibited a total editing efficiency of approximately 66%, with a diverse range of small to moderate deletions (−20 bp, −15 bp, −10 bp, and −5 bp) distributed around the predicted Cas9 cleavage site. In contrast, clone 3B2 demonstrated a higher overall editing efficiency (~74%) dominated by fewer, but larger, deletion events (most notably −25 bp and −4 bp). Model fit (R^2^) values for both clones were high (0.93 for 6A1 and 0.92 for 3B2), supporting robust decomposition and the accurate estimation of editing outcomes. The TIDE analysis of the clones indicated compound editing (i.e., biallelic) as potentially resulting in a greater impact on the target.

### 3.3. Disruption of scaRNA1 Results in Altered Expression

**Figure 4:**
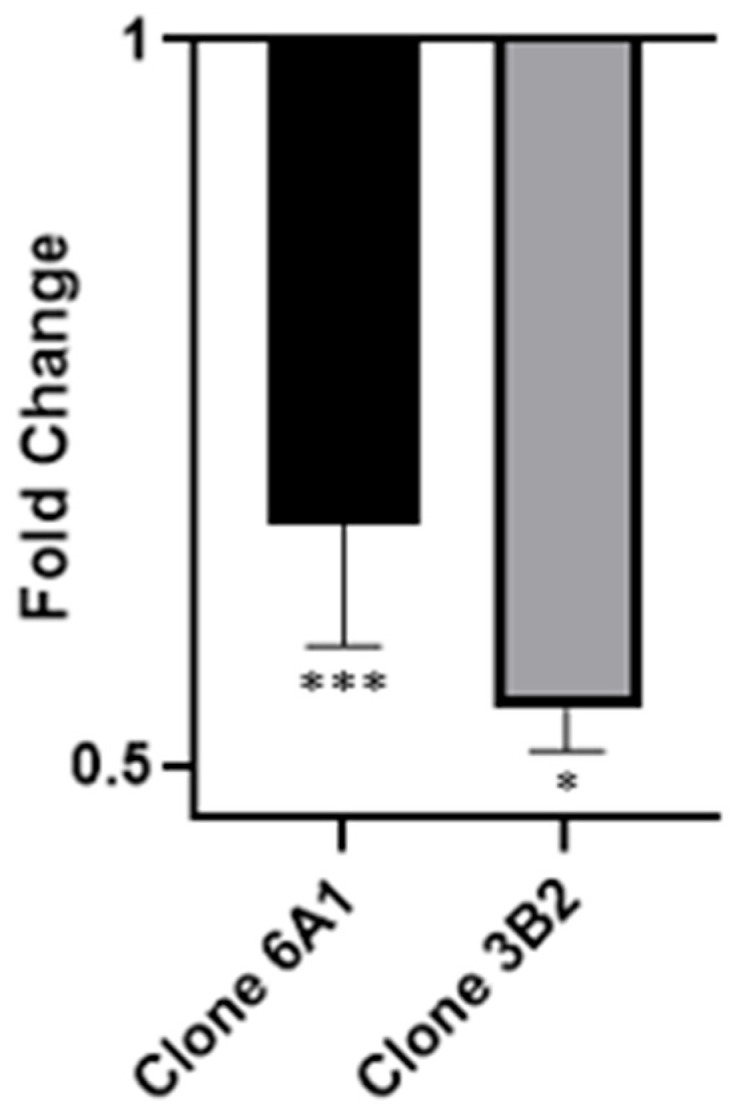
*ScaRNA1* expression change after CRISPR editing. *scaRNA1* expression levels were significantly reduced in clone 6A1 (*** *p* < 0.001, −1.67-fold change, n = 3) and 3B2 (* *p* < 0.05. −1.9-fold change, n = 2) relative to the control. The expression level of the *scaRNA1* host gene, *PPP1R8*, was not significantly altered.

To determine the impact of targeted CRISPR-Cas9 editing on scaRNA1 expression, we performed qPCR analysis on RNA from the clones 6A1 HEK293T and 3B2 HEK293T harboring CRISPR-induced indels within the scaRNA1 locus. The expression levels of scaRNA1 were quantified and normalized to the reference gene RN7SL. Specifically, scaRNA1 expression was reduced by approximately 40% in clone 6A1 and 47% in clone 3B2 (Figure 4) relative to the RNA from control cells.

### 3.4. Pseudouridylation Is Reduced Following CRISPR-Cas9 Editing of scaRNA1

**Figure 5:**
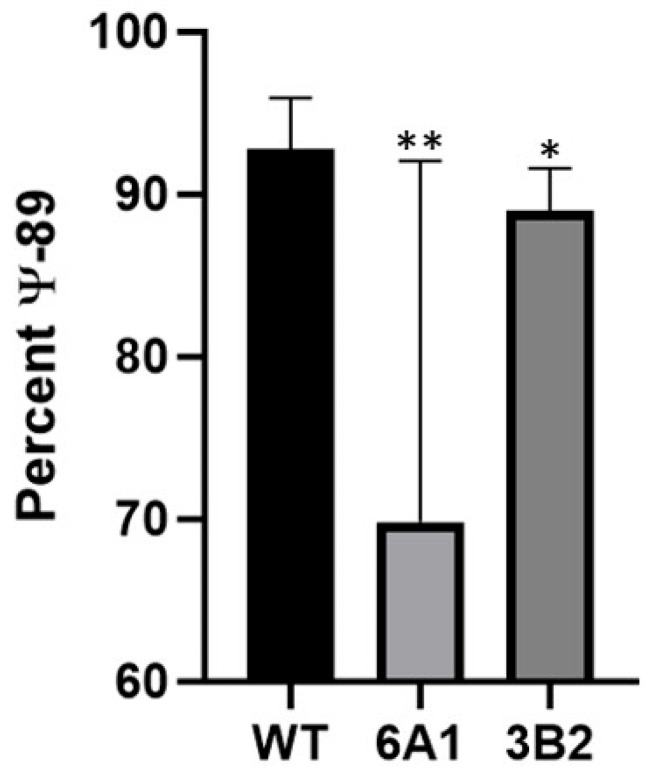
Average percentage of pseudouridylation at U89 in U2 snRNA following CRISPR-Cas9 editing of scaRNA1. Both clones had significant reductions in the percentage of pseudouridylation: clone 6A1 (70% ** *p* = 0.03; n = 5); clone 3B2 (88% * *p* < 0.04, n = 6) relative to the control (93%, n = 9).

To evaluate the effect of CRISPR-Cas9-mediated scaRNA1 disruption on site-specific RNA modification, pseudouridylation at uridine 89 (U89) in U2 snRNA was quantified using a CMC-based RT-qPCR assay. As shown in Figure 5, pseudouridylation levels were significantly reduced in the edited clone compared to wild-type (WT) controls.

Clone 6A1, which underwent dual-gRNA CRISPR editing of the scaRNA1 locus, showed a significant reduction in pseudouridylation compared to WT (Figure 5) These results confirm that targeted disruption of scaRNA1 reduces pseudouridylation at its known site on U2 snRNA, supporting its functional role in guiding site-specific RNA modification. We analyzed a second clone, 3B2, which showed a less robust impact on pseudouridylation. Pseudouridylation was reduced by 3%, which was still significant (*p* < 0.03, n = 6).

### 3.5. RNA-Seq

**Figure 6:**
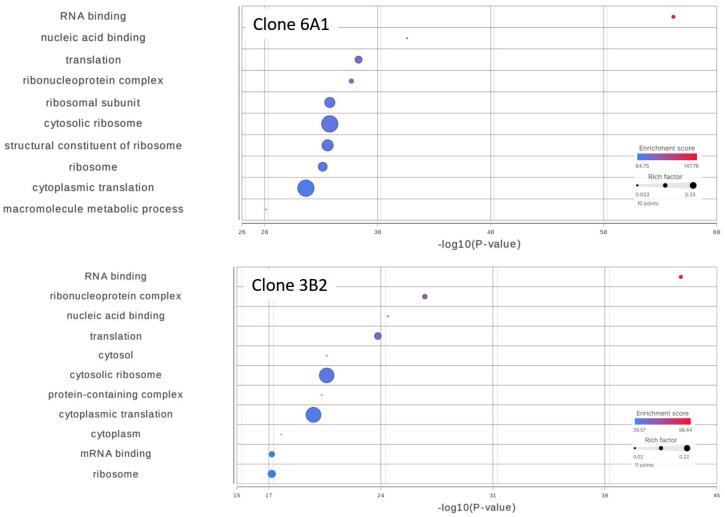
Analysis of mRNA isoforms with significant expression level changes after *scaRNA1* editing. Gene ontology (GO) enrichment analysis revealed highly significant over-representation of the “RNA-binding” molecular function category. Clone 6A1 and 3B2 had similar GO enrichment patterns.

Transcriptome sequencing performed independently on clone 6A1 and 3B2 revealed significant changes at the transcript level following the CRISPR-Cas9-mediated modification of *scaRNA1*. We identified a total of 293 genes for clone 6A1 and 220 genes for clone 3B2 with statistically significant changes in transcript isoform levels. Gene ontology (GO) enrichment analysis displayed a highly significant over-representation of the “RNA-binding” molecular function category (GO:0003723), with an enrichment score of 147.76, *p* = 6.77 × 10^−65^, and FDR step up = 1.47 × 10^−60^ for 6A1, and an enrichment score of 98.44, *p* = 1.77 × 10^−43^, and FDR step up = 3.84 × 10^−39^ for clone 3B2 (Figure 6). Notably, a great proportion of affected genes (134/293 for 6A1, 95/220 for 3B2) were functionally associated with RNA-binding activities, underscoring a profound impact of *scaRNA1* perturbation on RNA metabolism and post-transcriptional regulatory networks. Furthermore, significant transcript level changes were observed within several RNA-binding protein genes, indicating the extensive disruption of normal splicing mechanisms.

**Figure 7:**
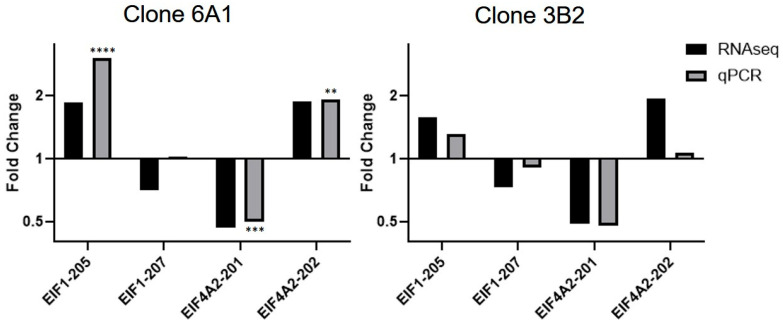
qPCR validation of transcripts predicted to have altered expression level by RNA-seq analysis. qPCR validation for each isoform was performed on RNA extracted from clone 6A1 and clone 3B2. EIF1 and EIF4A were chosen because they had a very high splicing index and belonged to the RNA-binding category highlighted by GO analysis. (** *p* < 0.01, *** *p* < 0.001, **** *p* < 0.0001). RNA-seq does not have a standard deviation because these values represent foldchange from RNA-seq data performed on single samples. qPCR values for clone 3B2 do not have a standard deviation because they were only run once as there was insufficient RNA for more analyses. They are shown to illustrate a similar trend of mRNA isoform alteration in both clones.

We validated mRNA isoform changes predicted by RNA-seq for two genes, EIF1 (isoforms 205 and 207) and EIF4A2 (isoforms 201and 202), using qPCR with primers designed for the amplification of specific mRNA isoforms (Figure 7). The pattern of isoform change was generally in agreement between RNA-seq and qPCR validation in both clones, 6A1 and 3B2.

## 4. Discussion

### 4.1. Functional Disruption of scaRNA1 Impairs Expression and Pseudouridylation Activity

The relationship between the editing outcome and functional impact was analyzed by TIDE analysis to characterize the indel profile of clone 6A1. Despite a moderate overall editing efficiency (~66%), 6A1 and 3B2 displayed a diverse set of deletions ranging from −20 to −5 base pairs. These indels likely disrupted essential structural elements required for scaRNA1 function. The significant reduction in Ψ89 pseudouridylation in the clones suggests that the composition of indels, not just their frequency, is a key determinant of functional outcome.

The CRISPR-Cas9 editing of the scaRNA1 locus in HEK293T cells resulted in both the molecular and functional disruption of the RNA. Clone 6A1 exhibited a 48% reduction in scaRNA1 expression compared to wild-type, as measured by RT-qPCR. The reduction in scaRNA1 expression by clone 3B2 was nearly 50%, which was modestly reduced by 5%, but still significant. Interestingly, despite the limited reduction in uridine 89 in RNA from clone 3B2, both clones demonstrated that scaRNA1 expression reduction alters mRNA splicing significantly and in a consistent manner.

These findings confirm that the CRISPR-Cas9 editing of scaRNA1 can effectively reduce RNA expression and disrupt its biological activity. These experiments also highlight the importance of considering indel structure when targeting noncoding RNAs, as small sequence alterations may significantly impact RNA-guided modification pathways. These results underscore the need for thoughtful guide RNA design and detailed molecular characterization when studying noncoding RNA functions through genome editing.

### 4.2. scaRNA1 Disruption Alters Alternative Splicing of RNA-Binding Genes

RNA sequencing analysis reveals that scaRNA1 disruption not only impacts direct targets but also influences broader post-transcriptional networks. In clone 6A1, RNA-binding proteins represent 46% (134/293) of transcripts with changed isoforms. The RNA-seq data for clone 3B2 exhibited striking similarity to that of 6A1 despite the more moderate impact on pseudouridylation, with 43% (95/220) of genes encoding for RNA-binding proteins.

This RNA-binding protein enrichment suggests that scaRNA1 disruption may propagate secondary effects through the destabilization of the splicing machinery itself. These findings collectively suggest that the targeted disruption of scaRNA1 critically alters the splicing of key regulatory genes, potentially impacting fundamental cellular processes and highlighting its significant role in RNA biology. Although we did not assess protein-level consequences, changes in isoform usage suggest possible effects on protein domain composition, localization signals, and regulatory motifs. Such alterations may disrupt RNA-binding protein functions and lead to systemic dysregulation of RNA processing pathways.

## 5. Limitations and Future Directions

Several limitations must be considered. First, the RNA-seq analysis was restricted to just two edited clones, and the low abundance of RNA from the second clone did not allow replicate PCRs. The data shown is from a single PCR run. However, the consistent outcome provides confidence in the assessment. Additionally, future studies need to include rescue experiments (re-expressing scaRNA1 in the clones) to confirm the cause and effect of reducing scaRNA1 expression. In addition, we plan to use animal model studies to define the full impact of scaRNA1 disruption on cellular tissue differentiation. Such efforts will deepen our understanding of how scaRNA1 loss contributes to developmental pathology and will inform potential therapeutic strategies targeting RNA-mediated regulatory pathways.

## 6. Biological and Clinical Significance

Previous work from our group demonstrated that scaRNA1 and other Cajal body-associated RNAs are significantly downregulated in right ventricular tissue of infants with Tetralogy of Fallot (TOF). This reduction correlated with aberrant splicing of cardiac development genes. In this study, CRISPR-Cas9 editing of scaRNA1 recapitulated key molecular defects, including reduced pseudouridylation and splicing alterations in RNA-binding proteins.

Notably, RNA-seq data from edited clones revealed the enrichment of splicing changes in RNA-binding protein genes, supporting the hypothesis that scaRNA1 supports a regulatory network essential for RNA metabolism and developmental fidelity. Disruption of this network may have far-reaching consequences, particularly in tissues with high splicing demands, such as the developing heart.

In summary, scaRNA1 plays a critical role in spliceosomal RNA modification and global splicing regulation. Its disruption alters the processing of key regulatory transcripts and may contribute to regulating cell specificity. These findings enhance our mechanistic understanding of RNA-guided splicing control and may inform future assessment of molecular genetic regulation of development.

## Figures and Tables

**Table 1:** CRISPR gRNA sequences and target sites in *scaRNA1*.

gRNA	Sequence	Strand	PAM	Cut Site in *scaRNA1* (bp)
gRNA1	AGTGTATCTTTACACACTGGGCT	Antisense	TGG	115
gRNA2	CACTCTGAGTTATAACTCAG	Sense	AGG	162

Note: Cut sites are estimated 3 bp upstream of the PAM, per SpCas9 targeting rules.

**Table 2:** PCR primers for *scaRNA1* target region.

Primer Name	Sequence (5′–3′)	Direction	Amplicon Size, bp
Forward Primer	GACAGGGTTGCCTAGTCTTAAT	Sense	990
Reverse Primer	CATACTAGAGATTTAGGGATTTCTTTGAGAGG	Antisense	

**Table 3:** Primers for quantification of scaRNA1, hostgene PPP1R8, EIF1, and EIF4A2 by qPCR.

Primer Name	Sequence (5′–3′)	Direction	Amplicon Size, bp
*RN7SL* Forward	ATCGGGTGTCCGCACTAAGTT	Sense	125
*RN7SL* Reverse	CAGCACGGGAGTTTTGACCT	Antisense	
*scaRNA1* Forward	GCACTTGATACTAACCGAGCTG	Sense	138
*scaRNA1* Reverse	AGTGTATCTTTACACACTGGGCT	Antisense	
*PPP1R8* Forward	CCCTCCCGGTTTACATCT	Sense	202
*PPP1R8* Reverse	ACTGTTGAGATCTATCAGGAAA	Antisense	
*EIF1 205* Forward	CTGCTGGCACTGAGGATTAT	Sense	
*EIF1 205* Probe	CAATACATACAACACAGCTGGCACCC	Antisense	
*EIF1 205* Reverse	ACGAATTAGGCCTAGCAAGAG	Antisense	253
*EIF1 207* Forward	CTGAAGGTTCATGGGTTTAAAAACCTC	Sense	
*EIF1 207* Probe	ACTTGGACTAGTGTAACTCCTTCATGCA	Sense	
*EIF1 207* Reverse	CAAGACTAGACAGCATGGCTCTT	Antisense	126
*EIF4A2 201* Forward	GTGCAACAAGTGTCTTTGGTTAT	Sense	
*EIF4A2 201* Probe	CTATATTCACAGAATTGGCAGAGG	Sense	
*EIF1 201* Reverse	CCACACCTTTCCTCCCAAAT	Antisense	103
*EIF4A2 202* Forward	AACTATATTCACAGGAGTCGATAGC	Sense	
*EIF4A2 202* Probe	CAGTTGGTGACGAGATGGCACTCA	Sense	
*EIF1 202* Reverse	CAGTGTGCTGTTTCGCTTATG	Antisense	105

**Table 4:** Primers for quantification of pseudouridylation by RT-qPCR.

Primer Name	Primer Sequence	Description
*HuU2*-Ψ89UpF-CN-CMC	CTGATACGTCCTCTATCCGA	Upstream Forward
*HuU2*-Ψ89DnF-CN-CMC	TGGAGCAGGGAGATGGAATAGG	Downstream Forward
*HuU2*-Ψ89shR-CN-CMC	TACTGCAATACCAGGTCGATGCGT	Shared Reverse

## Data Availability

The data used and/or analyzed during the current study are available from the corresponding author on reasonable request.
